# Highly wearable cuff-less blood pressure and heart rate monitoring with single-arm electrocardiogram and photoplethysmogram signals

**DOI:** 10.1186/s12938-017-0317-z

**Published:** 2017-02-06

**Authors:** Qingxue Zhang, Dian Zhou, Xuan Zeng

**Affiliations:** 1 Departpment of Electrical Engineering, University of Texas at Dallas, 800 W Campbell Rd, Richardson, TX 75080 USA; 2 Department of Microelectronics, Fudan University, 220 Handan Rd, Shanghai, 200433 China

**Keywords:** Wearable computers, Blood pressure, Heart rate, Photoplethysmogram, Electrocardiography, Pulse transit time, Machine learning

## Abstract

**Background:**

Long-term continuous systolic blood pressure (SBP) and heart rate (HR) monitors are of tremendous value to medical (cardiovascular, circulatory and cerebrovascular management), wellness (emotional and stress tracking) and fitness (performance monitoring) applications, but face several major impediments, such as poor wearability, lack of widely accepted robust SBP models and insufficient proofing of the generalization ability of calibrated models.

**Methods:**

This paper proposes a wearable cuff-less electrocardiography (ECG) and photoplethysmogram (PPG)-based SBP and HR monitoring system and many efforts are made focusing on above challenges. Firstly, both ECG/PPG sensors are integrated into a single-arm band to provide a super wearability. A highly convenient but challenging single-lead configuration is proposed for weak single-arm-ECG acquisition, instead of placing the electrodes on the chest, or two wrists. Secondly, to identify heartbeats and estimate HR from the motion artifacts-sensitive weak arm-ECG, a machine learning-enabled framework is applied. Then ECG-PPG heartbeat pairs are determined for pulse transit time (PTT) measurement. Thirdly, a PTT&HR-SBP model is applied for SBP estimation, which is also compared with many PTT-SBP models to demonstrate the necessity to introduce HR information in model establishment. Fourthly, the fitted SBP models are further evaluated on the unseen data to illustrate the generalization ability. A customized hardware prototype was established and a dataset collected from ten volunteers was acquired to evaluate the proof-of-concept system.

**Results:**

The semi-customized prototype successfully acquired from the left upper arm the PPG signal, and the weak ECG signal, the amplitude of which is only around 10% of that of the chest-ECG. The HR estimation has a mean absolute error (MAE) and a root mean square error (RMSE) of only 0.21 and 1.20 beats per min, respectively. Through the comparative analysis, the PTT&HR-SBP models significantly outperform the PTT-SBP models. The testing performance is 1.63 ± 4.44, 3.68, 4.71 mmHg in terms of mean error ± standard deviation, MAE and RMSE, respectively, indicating a good generalization ability on the unseen fresh data.

**Conclusions:**

The proposed proof-of-concept system is highly wearable, and its robustness is thoroughly evaluated on different modeling strategies and also the unseen data, which are expected to contribute to long-term pervasive hypertension, heart health and fitness management.

## Background

Blood pressure (BP) is a key health indicator to diagnose and control hypertension, which impacts over 35% of people worldwide, relates to cardiovascular, circulatory and cerebrovascular diseases, and causes 12.8% of the total of all deaths [1]. Since BP fluctuates over time, BP should not be measured only at specified times and circumstances. However, the traditional BP measurement approaches are unsuitable for long-term ubiquitous applications, such as the invasive catheterization method and the noninvasive cuff-based oscillometry method [2], which are both time consuming and of a poor wearability.

Nowadays, wearable computers are paving a promising way for ubiquitous BP monitoring by providing convenient and long-term out-of-clinic measurements. Wearable cuff-less BP monitors are usually created leveraging mounting evidence that the pulse transit time (PTT) is reversely related to the BP [2]. When building the BP model, the PTT is usually treated as a denominator, or as a numerator but with a negative coefficient, depending on the underlying assumptions [2]. To measure the PTT, i.e., the time delay for the pressure wave to propagate between two arterial sites, one popular method is based on two signals, i.e., electrocardiography (ECG) and photoplethysmogram (PPG). The former one is the electrical signal generated by the heart, while the latter one measures fluctuations in the blood volume which are caused by the mechanical pressure pulse and thus changes later than the electrical ECG wave. Currently, the ECG signal is usually measured using a single- or multiple-lead configuration, referring to or modified from the traditional standard 12-lead configuration which can provide strong ECG signals with highly distinguishable morphologies [2, 3]. Nevertheless, these electrodes placement methods may have some limitations in long-term applications, e.g., the chest electrodes placement may be uncomfortable especially when sweating, and the two wrists configuration may be still inconvenient since additional wires or separate devices are inevitable. Likewise, the PPG sensor is usually placed on the chest which may be uncomfortable, or on the finger where more challenges may be posed to the integration of PPG and ECG sensors [4].

In this paper, we propose a single-arm blood pressure monitoring system, which allows for placing the PPG sensor and the ECG electrodes all on the left upper arm, to enable long-term daily applications which have critical requirements on the wearability and comfortableness. Since we put both ECG signal and reference electrodes on the left upper arm which form a non-standard single-lead configuration for super wearability, the potential difference between these two close electrodes due to the heart electric propagation is so small that it is highly challenging to obtain a clear single-arm-ECG signal. By creating a customized hardware prototype and placing the reference electrode on the top side of the left upper arm and the signal electrode on the bottom side to maximize the distance between these two electrodes, the weak single-arm-ECG signal is successfully acquired, which owns an amplitude much lower than those measured by the standard or modified traditional lead configurations mentioned above, but has a morphology still distinguishable. The single-arm-PPG signal is also acquired by placing the sensor close to the ECG electrodes for a good wearability. Afterwards, to recognize the heartbeats from the weak ECG signal with many interferential spikes induced by motion artifacts and electromyography (EMG) noise, a machine learning-enabled framework is introduced [5]. Based on the identified heartbeats in the ECG signal, the heartbeat pairs in the PPG signal are then determined to obtain the PTT measurements which will be used to build the systolic BP (SBP) model (we take special interest in SBP monitoring in this study).

Recent investigations have also introduced the heart rate (HR) information to enhance the robustness of the blood pressure models, based on the consideration that the cardiac output flow usually increases with HR, and thus SBP would increase with HR if assuming the arteries is purely resistive [6, 7]. In our study, we choose the PTT&HR-based SBP model to estimate the SBP, with the HR information estimated from the ECG heartbeats identified. Meanwhile, we are also interested in how much contribution the HR information can bring to the robustness improvement of the SBP model. Actually, the PTT-SBP relationship has been investigated by large amounts of studies over many decades, but these models only represent some facets of the physiology rather than all know behaviors [2]. A quantitative evaluation on the effectiveness of new variables in SBP model improvement is nontrivial considering the underlying complex blood pressure generation and propagation mechanisms. Therefore, we take into account ten SBP models including both PTT&HR-SBP and PTT-SBP models and give a comparative analysis.

These SBP models are firstly tuned using the training data, and then evaluated on the unseen fresh testing data to show the generalization ability of the tuned models. Therefore, the algorithm had been set before the testing performance evaluation stage and not changed during evaluation. Moreover, the SBP estimates based on the chest-ECG/arm-PPG signals are also obtained to show the feasibility to replace the strong but inconvenient chest-ECG with the weak arm-ECG, to enable the highly wearable SBP monitoring. Besides, the participants were asked to perform exercise during some signal periods in data collection to introduce more stress to the signal quality. The exercise stress can not only perturb the SBP to a larger range to increase the diversity, but also introduce more motion artifacts and heart rate variability to the weak arm signals towards practical applications. Experimental results show that the HR can be robustly estimated from the weak single-arm-ECG signal, and the PTT&HR-SBP with HR enhanced significantly outperform the PTT-SBP models and can be well generalized to the unseen data. Therefore, the single-arm-ECG signal can be a highly convenient and effective alternative to the chest-ECG signal, to enable robust long-term SBP monitoring applications together with the single-arm-PPG signal.

## Methods

### System overview

**Fig. 1:**
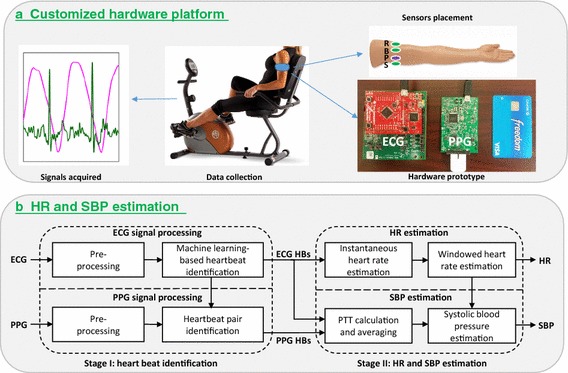
The proposed system for wearable cuff-less SBP and HR monitoring with single-arm-ECG and PPG signals. *R*/*B*/*S* represent the reference/bias/signal electrodes used for single-lead ECG signal measurement, respectively, *P* corresponds to the reflective PPG sensor; *PTT* pulse transit time, *HB* heartbeat, *HR* heart rate, *SBP* systolic blood pressure

The proposed wearable cuff-less blood pressure and heart rate monitoring system is illustrated as Fig. 1, where the top part (Fig. 1a) shows the customized hardware platform for single-arm-ECG and PPG signals acquisition, and the bottom part (Fig. 1b) gives the flow of the HR and SBP estimation algorithms.

### Customized hardware platform

The customized hardware platform comprises two parts, i.e., the ECG signal [5] and PPG signal acquisition subsystems, as shown in the right part of Fig. 1a. The former one includes a TI ADS1299EEG-FE evaluation board (blue one) which is equipped with an ADS1299 24-bit analog-to-digital converter (ADC) for low voltage bio-potential measurement, and a TI TivaTM C series LaunchPad [8] (red one) which includes an ARM Cortex M4 microcontroller (MCU) to send commands to the ADC, read the measurements from the ADC via the SPI port and give the data to a PC via the USB port. The latter one includes a TI AFE4490SPO2 evaluation board [9] (blue one) which is equipped with an LED transmit section to generate the red or infrared light to illuminate the skin, and a low-noise receiver channel with a 22-bit ADC to measure the time varying light absorption by the tissue to reflect the changes in the blood volume. There is an MSP430F5529IPN MCU embedded on this board to configure the ADCs, fetch the data from the receiver ADC via the SPI port, and send the data to the PC by the USB port. After removing the components only for evaluation purposes and adding a wireless communication module, the proposed prototype can be conveniently used in long-term wearable applications.

The ECG and PPG sensors placement on the left upper arm is illustrated in the right part of Fig. 1a, where the circles labeled as R/B/S represent the reference/bias/signal electrodes used for single-lead ECG signal measurement, respectively, and the circle labeled as P corresponds to the reflective PPG sensor [10] with the LEDs and photodiode embedded for PPG signal acquisition. The proposed sensors placement method is highly convenient, since it prevents attaching the ECG electrodes to the chest, or to multiple separate body sites such as two wrists plus one finger [4]. Moreover, the ECG electrodes and the PPG sensor can be integrated into a single-arm band, to further enhance the wearability in long-term daily applications.

### Data acquisition protocol

The customized platform was used for single-arm-ECG and PPG signals acquisition, with a sampling rate of 500 and 128 Hz, respectively. The chest-ECG signal is collected at the same time for comparison purpose. A higher sampling rate for ECG is based on the consideration that it is used not only for PTT but also for HR estimation. The data collection was performed on ten participants (age: 35 ± 14; Weight: 68 ± 13 kg; height: 168 ± 7 cm; Gender: 7 males and 3 females) to demonstrate our proof-of-concept system. Informed consent was obtained from all individual participants included in the study. For each subject, the data included two sessions collected on the same day using the same data acquisition protocol, i.e., a 26-min training session used to train the algorithms, such as the heartbeat identification classifier and the SBP models, and another 26-min testing session, to evaluate the generalization ability to the unseen data of the trained algorithms. Each subject was asked to sit on an IMPEX MARCY ME-709 recumbent exercise bike [11] with armrests. Each session took 26 min, including 13 2-min trials belonging to three parts, i.e., part I (trial 1), part II (trial 2–11) and part III (trial 12–13). During part I and III, the subject stayed still, and during each trial in part II, the subject rode the bike in the first minute and stayed still in the second minute. The exercise was introduced to perturb the SBP to a larger range referring to protocol used in [12], such that SBP model can be trained and tested both over a larger range of SBP. Both cuff-based SBP measurement and ECG/PPG-based SBP estimation were performed in the same time duration, i.e., the second minute of each trial when subjects stayed still. We used an ambulatory blood pressure monitor CONTEC ABPM50 [13] to measure the reference SBP, which consumes about one minute to report one measurement result. Correspondingly, we used the ECG/PPG signals in the second minute of each trial for averaged PTT and HR estimation, which were then used in the SBP model training and testing. In this way, we can guarantee the synchronization between the reference and the estimates. Considering that the reference SBP was measured when the subjects stayed still and put their forearms on the armrests of the exercise bike, the reference SBP can be robustly measured by the ambulatory blood pressure monitor. Therefore, all the data has been used in our analysis. One thing worth noting is that the exercise stress can also introduce more motion artifacts and heart rate variability to the weak arm signals, to take into account more affecting factors in practical application scenarios.

### Signal pre-processing

**Fig. 2:**
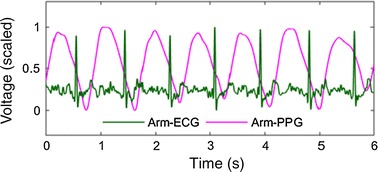
Arm-ECG/PPG signals, with the amplitude both scaled to be between 0 and 1 for good readability (Arm-ECG signal is actually much weaker than arm-PPG, which will be further analyzed later)

As shown in the left part of Fig. 1b, the acquired raw ECG signal is pre-processed by a Butterworth bandpass filter with a lower and upper cutoff frequencies of 2 and 30 Hz, respectively, to remove the baseline wander and powerline interference and suppress the motion artifacts. The raw PPG signal is processed by a Butterworth filter with cutoff frequencies of 0.5 and 8 Hz, respectively, and then it is resampled to 500 Hz to own a same time resolution as ECG. In Fig. 2, an example of the filtered single-arm-ECG and PPG signals is given, which shows ECG and PPG pulses. Besides, it is found that the exercise usually more or less affects the signal quality and distorts the morphologies, which will be further analyzed in the results section.

### ECG-based heartbeat identification

ECG-based heartbeat locations are used in both PTT calculation and HR estimation as shown in Fig. 1b. To robustly identify heartbeats from the weak arm-ECG signal, our previously reported machine learning-enabled framework (MLEF) is applied [5], which can effectively identify corrupted heartbeats and robustly estimate the heart rate from the wrist-ECG signals based on the support vector machine (SVM) classifier, even with an SNR as low as −7 dB. This heartbeat identification framework includes the following four steps:

Step 1: ECG stream auto-segmentation for heartbeat candidate generation.

Step 2: Feature extraction for each heartbeat candidate.

Step 3: SVM model training on the training data; or SVM model testing on the fresh testing data.

Step 4: Get identified high confident heartbeats.

In this first step, an adaptive threshold is generated based on the time-varying fluctuation of the signal. When there is a larger peak-to-peak voltage in a time window (20 s) due to motion artifacts, the vertical fluctuation of the real heartbeats is also increased, and vice versa. Therefore, we introduce an extra item to adaptively adjust a pre-defined fixed threshold to track the signal fluctuation, such that wherever possible, the real heartbeats can be selected as the heartbeat candidates, to guarantee a high sensitivity. Meanwhile, many motion artifacts-induced interferential spikes are also selected, resulting in a low precision, therefore, the next steps will further identify high confident heartbeats from the heartbeat candidates.

In the second step, ten critical motion artifacts-tolerant features are extracted from multiple domains for each candidate, include *R angle* (angle of the R peak)*, S angle* (angle of the S valley)*, RS Diff* (voltage difference between the R peak and the S valley)*, R Symmetry* (the symmetry of the R peak)*, S Symmetry* (the symmetry of the S valley)*, SKNS* (skewness of the R peak region)*, VAR* (variance of the R peak region)*, RMS* (root mean square of the R peak region)*, alpha*-*3* (angle of the slop of the third sample on the left side of the R peak), and *alpha 2* (angle of the slop of the second sample on the right side of the R peak). These features are selected from twenty-six raw features by a sparse SVM which can effectively push the non-significant features towards zero [5].

In the third step, an SVM model is trained firstly on the training data, and then tested on the fresh testing data for heartbeat identification. The SVM can constructs a hyperplane to effectively classify the instances into different groups. To train an SVM model, a constrained quadratic optimization problem is solved. The objective function is composed of two parts, i.e., the regularization part ($\frac{1}{2}w^{2}$) and the loss caused by misclassified instances ($C\mathop \sum \nolimits_{i}^{M} \xi_{i}$), as shown in (1), where $w$ is a weight vector to be sought, $C$ is a tradeoff parameter between maximization the separation margin and the minimization of the classification error ($C$ is chosen as 1 as suggested by [14]), and $\xi_{i}$ is the nonnegative slack variables to penalize the misclassified instances (1 to *M*). There are two constraints shown as (2, 3), where $y_{i}$ is the class label of the instance $x_{i}$, $\varPhi (x_{i} )$ is the kernel function to transform the instance $x_{i}$ to the kernel space, and $b$ is the bias to be sought. A linear kernel is chosen to lower the computation load for wearable applications.

1 $$\mathop {\hbox{min} }\limits_{w,b} \frac{1}{2} \Vert w^{2}\Vert + C\mathop \sum \limits_{i = 1}^{M} \xi_{i}$$

2 $$s.t.\;\; y_{i} \left( {w^{T} \cdot {\varPhi }\left( {x_{i} } \right) + b} \right) \ge 1 - \xi_{i} , \quad \forall x_{i}$$

3 $$\xi_{i} \ge 0$$

After introducing the Lagrange multipliers $\alpha_{i}$, we now have the dual problem as shown in (4–6), where $K\left( {x_{i} ,x_{j} } \right) = {\varPhi }\left( {x_{i} } \right) \cdot {\varPhi }\left( {x_{j} } \right)$ representing the inner production operation between two instances in the transformed space. This dual problem can then be solved by a sequential minimal optimization method [14].

4 $$\mathop {\hbox{max} }\limits_{{\alpha_{i} }} \mathop \sum \limits_{i = 1}^{M} \alpha_{i} - \frac{1}{2}\mathop \sum \limits_{i = 1}^{M} \mathop \sum \limits_{j = 1}^{M} \alpha_{i} \alpha_{j} y_{i} y_{j} K\left( {x_{i} ,x_{j} } \right)$$

5 $$s.t. \mathop \sum \limits_{i = 1}^{M} \alpha_{i} y_{i} = 0$$

6 $$C \ge \alpha_{i} \ge 0, \quad \forall i = 1, \ldots ,M$$

Based on the learned $\alpha_{i}$ and support vectors on the training data, we now can predict a label $\overline{y}$ for any $x$ using the following decision function in (7) on the fresh testing data, which means for each heartbeat candidate in the testing session, this function can predict whether it is a heartbeat or motion artifacts-induced interferential spikes. The identified ECG-based heartbeats from the weak arm-ECG stream will then be used in PPG-based heartbeats determination and also heart rate estimation.

7 $$\overline{y} = sign\left( {\mathop \sum \limits_{i = 1}^{M} \alpha_{i} y_{i} K\left( {x_{i}^{T} ,x} \right) + b} \right)$$

In the fourth step, after we run the SVM classification to identify the high confident heartbeats, we can get all high confident heartbeats from the heartbeat candidates which include both real heartbeats and motion artifacts-induced interferential heartbeat-like spikes. The identified ECG heartbeats are then used for heart rate estimation, and also PPG heartbeat identification.

One thing worth noting is that the SVM-based heartbeat identification algorithm can be run in real-time. After training the SVM classifier based on all training data, the trained SVM model can be applied to each heartbeat candidate in the fresh testing data to predict whether it is a real heartbeat or a motion-artifacts-induced interferential spike.

### PPG-based heartbeat identification

**Fig. 3:**
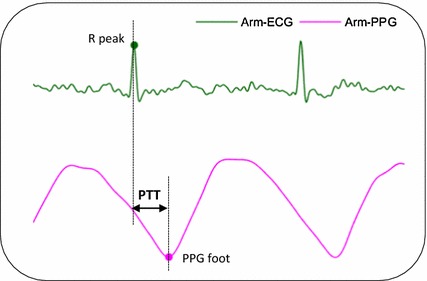
Pulse transit time measured with ECG and PPG signals (This illustration of PTT is based on arm-ECG/PPG signals)

PPG-based heartbeat arrives later than the ECG-based heartbeat, as shown in Fig. 3, where a pink dot corresponds to a PPG waveform foot and has been used in many works to represent the PPG-based heartbeat occurrence time [15, 16], and a green dot corresponds to the R peak of an ECG pulse and represents the ECG-based heartbeat occurrence time. Correspondingly, in our algorithm as shown in Fig. 1b, the minimum point between two adjacent R peaks are identified as the PPG-based heartbeat locations.

### Heart rate estimation

As illustrated in the top right part of Fig. 1b, after calculating the instantaneous heart rate (denoted as IHR) based on the identified ECG-based heartbeats, the windowed heart rate (denoted as HR or windowed HR) estimates are then achieved by averaging the IHR estimates oven each time window, with the window corresponding to the second minute in each 2-min trial during which the reference SBP is measured. The performance of the windowed HR in the testing session will be evaluated in term of mean absolute error (MAE) and root mean square error (RMSE), with a unit of beats per min (BPM).

### Pulse transit time

Pulse transit time reflects the time delay between the pressure pulse flows from the proximal to the distal arterial sites [2]. When using ECG and PPG to estimate the PTT as shown in Fig. 3, the proximal arterial site usually refers to the thoracic aorta and the corresponding PTT start time is approximately measured as the ECG heartbeat R peaks, while the distal site often means the skin surface where the PPG signal is collected and its waveform foot gives the PTT end time. Therefore, the PTT can be obtained by subtracting the PTT start time from the PTT end time, i.e., the time delay between the R peak in the ECG signal and the waveform foot in the PPG signal, as shown in (8) where $i$ is the *i-th* $PTT$ to be estimated. The PTT values measured in the second minute of each trial are then averaged to obtain the window-based PTT estimates.

8 $$PTT_{i} = PPG_{i}^{foot} - ECG_{i}^{Rpeak}$$

However, although the above method for PTT estimation has been used in many works for convenient and simplicity purpose, the measurement actually includes another extra item, i.e., the pre-ejection period (PEP) which corresponds to the aortic valve opening time and usually makes the PTT measured significantly larger than its real value [17]. To measure PEP for SBP model improvement, an additional signal usually needs to be acquired, such as the impedance cardiography (ICG) or the phonocardiogram (PCG) [18], which inevitably causes extra hardware burden and impacts the wearability. Instead of measuring PEP to improve the PTT estimate, another strategy for SBP model enhancement is introducing HR to the original PTT-SBP model to form a new PTT&HR-SBP model, leveraging the correlation between HR and SBP [6]. Actually, introducing HR to the SBP model is natural since the HR information is already carried by the ECG signal and can be robustly measured by appropriate algorithms, such as MLEF in our study, without adding extra signal acquisition hardware.

### Blood pressure estimation

The PTT&HR-SBP model is chosen for SBP estimation considering both PTT and HR are correlated with SBP. Meanwhile, we also implemented PTT-SBP models for comparison purpose, which include a bunch of modeling strategies based on different assumptions, such as the linear, quadratic and exponential equations. In total, ten SBP models are evaluated as shown in Table 1, and a thorough comparative analysis based on experimental results will be given in the next sections to show that PTT&HR-SBP models are superior to PTT-SBP models.

**Table 1:** Ten blood pressure models for comparative analysis

No.	Equation	HR information
1	*SBP* = *a* ln (*PTT*) + *b*	w/o
2	$SBP = a \; PTT^{ - 1} + b$	w/o
3	$SBP = a\; PTT + b$	w/o
4	$SBP = a \;PTT^{2} + b\;PTT + c$	w/o
5	$SBP = a\; PTT^{2} + b$	w/o
6	$SBP = a \;e^{b PTT}$	w/o
7	$SBP = a\; PTT^{ - 2} + b$	w/o
8	$SBP = a \;PTT^{ - 2} + b\; HR^{ - 2} + c$	w/
9	$SBP = a\;\ln ( {PTT} ) + b\;\ln ( {HR}) + c$	w/
10	$SBP = a\; PTT + b\; HR + c$	w/

In Table 1, the listed ten blood pressure models not only cover SBP models based on the linear, quadratic and exponential assumptions, but also include SBP models with or without HR information embedded [2, 3, 6, 7, 17–19]. These models are based on different mechanisms and deduction processes. For example, in model 2, the PTT is reversely related to the SBP since the time delay for the mechanical pressure wave to propagate between the proximal and the distal sites is usually reduced with a higher SBP, and vice versa [2]. In model 7, the relationship between SBP and PTT is demonstrated based on the combined action of the pulse wave and the energy of wave (kinetic and the gravitational potential energy) [18]. In model 10, the embedding of HR is based on the consideration that the cardiac output flow usually increases with HR, and thus SBP would increase with HR if assuming the arteries is purely resistive [6]. More details of these models can be found in [2, 3, 6, 7, 17–19].

There are two things worth noting about the SBP models given in Table 1. Firstly, although the PTT is usually reversely correlated with SBP [2], it can be either used as a denominator, or as a numerator but with a negative coefficient fitted. For example, in the model 2 and model 3, PTT is used as a denominator and a numerator (corresponding denominator is 1), respectively. Secondly, these models may have different mechanisms, or may be tested on diverse scenarios in previous studies, such as location of sensors and subjects of different ages, however, they are all built on the fact that PTT is correlated with SBP (or HR is also correlated with SBP), and the model coefficients are also tailored (tuned) to each subject for better performance as suggested in [2].

To evaluate the generalization ability of the trained SBP models to the unseen data, the SBP models fitted on the data in the first session (training session) is tested on the unseen data in the second session (testing session). Both training and testing performance is given in terms of many different criterion, including Bland–Altman plot [20], mean error (ME) ± standard deviation (STD), MAE and RMSE.

## Results

In this section, both the proposed hardware prototype and the HR/SBP estimation algorithms are evaluated in detail, according to the signal processing flow shown in Fig. 1.

### Signals acquired

**Fig. 4:**
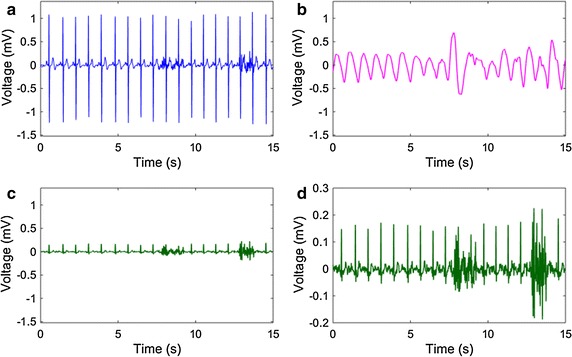
An example of the signal segments acquired including chest-ECG (**a**), arm-PPG (**b**) and arm-ECG (**c**, **d**), showing that (1) the weak arm-ECG has an amplitude only around 10% of that of chest-ECG, and (2) the arm-ECG is so sensitive to motion artifacts and EMG noise that advanced algorithms are necessary for robust heartbeat identification and heart rate estimation

The signals acquired by the proposed hardware prototype are given in Fig. 4. In Fig. 4a–c, three signals, i.e., chest-ECG, arm-PPG and arm-ECG, are compared in terms of several aspects including signal morphology and amplitude. A zoomed in version of the arm-ECG is also given in the bottom right part (Fig. 4d) to show the details. There are several interesting observations from these illustrations. Firstly, compared with the chest-ECG signal with the electrodes placed close to the heart, the arm-ECG signal has a much lower amplitude (around 10% of that of the chest-ECG signal in this example). This is due to the fact that the arm-ECG electrodes are put not only further from the heart, but also have a small relative distance since they are constrained by the same arm band. Actually, when the electrodes are not close to the heart, the distance between the reference and the signal electrodes is the key factor to be able to observe the ECG signal. In our study, by placing the reference electrode on the top side of the left upper arm and the signal electrode on the bottom side to maximize the distance between these two electrodes, a distinguishable heartbeat wave is finally observed as shown in Fig. 4d.

Secondly, some portion of the arm-PPG signal is of a clear morphology, indicating that the reflected LED light is quasi-periodically related to the amount of the blood flowing through the arm tissue under the PPG sensor. Thirdly, both the arm-ECG and arm-PPG signals are sensitive to the motion artifacts and EMG noise. This is due to the fact that the arm is almost all surrounded by muscles and even slight body movements usually relate to random stretching of these muscles, which may cause worse sensor-skin contact and thus induce many artifacts and noise to the signals. Even the signals without riding the bike are chosen to estimate the SBP, there is still considerable amounts of motion artifacts and EMG noise since the body is usually not strictly still, posing more challenges to the following heartbeat identification process.

### Heartbeat identification and heart rate estimation

The arm-ECG-based heartbeat locations (R-peaks) are firstly identified by MLEF and then the arm-PPG-based ones (PPG feet) are obtained by searching the minimum point in the arm-PPG signal between two adjacent R-peaks. Afterward, the PTT are estimated with the recognized R-peaks and PPG feet, which will be analyzed later. Meanwhile, the instantaneous heart rate information is obtained from the arm-ECG signal, denoted as IHR@arm-ECG. The IHR@arm-PPG is estimated at the same time for comparison purpose. Besides, to calculate the ground truth IHR, the heartbeats are detected from the strong chest-ECG signal by a simple threshold method and manually checked by us to guarantee the correctness. The obtained ground truth IHR is denoted as IHR@chest-ECG.

**Fig. 5:**
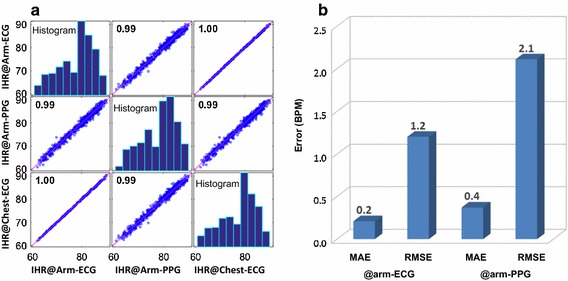
Heart rate estimation performance: **a** correlation matrix of estimated IHR@arm-ECG, estimated IHR@arm-PPG and ground truth IHR@chest-ECG, in the testing session of subject 1 (unit of IHR: beats per min, i.e., BPM); **b** performance of windowed HR in the testing session averaged over all subjects. *MAE* mean absolute error, *RMSE* root mean square error

The correlation matrix of the IHR estimates in the testing session of one subject using these three approaches is illustrated in Fig. 5a. In this example, a correlation as high as 1.00 between the IHR@arm-ECG and the IHR@chest-ECG indicates that the ECG heartbeat locations in the weak arm-ECG are successfully identified by the MLEF and thus can be used for robust IHR estimation. On the other hand, the IHR@arm-PPG owns a lower (0.99) correlation with the ground truth IHR@chest-ECG, which indicates that the time varying blood pressure often fluctuates the propagation time the mechanical pulse takes to arrive the tissue under the arm-PPG sensor and thus impacts the IHR estimation. Therefore, we choose the IHR@arm-ECG to get the windowed HR (averaged over the second minute of each trial) for SBP model enhancement.

The performance of windowed HR in the testing session averaged over all subjects is given in Fig. 5b, where both arm-ECG and arm-PPG-based HR are compared with the ground truth HR@chest-ECG. The HR@arm-ECG corresponds to an MAE of 0.21 BPM and a RMSE of 1.20 BPM, while HR@arm-PPG has higher errors, i.e., an MAE of 0.37 BPM and a RMSE of 2.11 BPM. The results are consistent with the IHR correlation matrix and further indicate that the arm-ECG can be a more robust alternative to chest-ECG in wearable applications.

### Correlation among PTT, HR and SBP

**Fig. 6:**
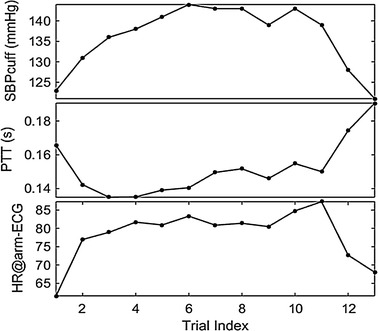
Trend of SBPcuff, PTT and HR@arm-ECG, corresponding to the training session of subject 1

After obtaining the PTT and HR with the identified heartbeats in the arm-ECG and PPG signals, an example of their correlation with the reference SBPcuff is illustrated in Fig. 6, based on the data in the training session of one subject. Specially, at the beginning of this session (trial 1), a large PTT relates to a low SBP. Afterwards, the SBP gradually increases due to the interventions (exercise) and then stays at a high level (trial 2–11). The PTT also gradually increases, but it does not steadily stay at a high level like the SBP. Finally, at the end of this session (trial 12–13), PTT increases and the SBP decreases over time.

As shown in Fig. 6, both PTT and HR are correlated with SBP, which is consistent with previous studies [6, 7] that showed the introduction of HR can contribute to the improvement of the SBP model. Meanwhile, we also want to quantitatively evaluate the contribution of the HR information in SBP model improvement, therefore, the SBP estimation performance for both PTT&HR-SBP and PTT-SBP models is illustrated later.

### Blood pressure estimation

The PTT&HR-SBP models 8–10 are firstly tuned on the training data, and then tested on the unseen fresh testing data as shown in Table 2. We choose the worst case performance model 10 among these three models, to fairly illustrate the generalization ability of the PTT&HR-SBP models when introducing the HR information. Based on arm-ECG and arm-PPG signals, the blood pressure estimation performance is 1.63 ± 4.44, 3.68 and 4.71 mmHg in terms of ME ± STD, MAE and RMSE, respectively. The estimation error is below $5 \pm 8$ mmHg in terms of ME ± STD, which meets the advancement of medical instrumentation (AAMI) standard [21].

**Table 2:** A summary of the testing performance of ten SBP models

No.	With arm-ECG and arm-PPG				With chest-ECG and arm-PPG			
	ME	STD	MAE	RMSE	ME	STD	MAE	RMSE
1	1.17	6.00	4.73	6.09	0.96	5.97	4.66	6.02
2	1.58	6.62	5.08	6.78	1.46	6.60	5.01	6.74
3	0.73	5.88	4.58	5.90	0.59	5.86	4.54	5.87
4	0.29	6.15	4.82	6.14	0.02	6.33	4.96	6.30
5	0.85	5.85	4.59	5.89	0.68	5.86	4.57	5.88
6	0.74	5.88	4.59	5.90	0.61	5.86	4.55	5.86
7	0.79	5.95	4.65	5.98	0.68	5.91	4.60	5.93
*8*	*1.59*	*4.44*	*3.69*	*4.70*	*1.58*	*4.41*	*3.62*	*4.67*
*9*	*1.62*	*4.43*	*3.67*	*4.70*	*1.61*	*4.41*	*3.61*	*4.67*
*10*	*1.63*	*4.44*	*3.68*	*4.71*	*1.61*	*4.42*	*3.62*	*4.69*

Furthermore, the SBP estimation performance based on the chest-ECG and arm-PPG is also given in Table 2. The results show that the estimation performance is very close to that based on the arm-ECG and arm-PPG. For example, the ME ± STD, MAE and RMSE for model 10 based on the chest-ECG and arm-PPG are 1.61 ± 4.42, 3.62 and 4.69 mmHg, respectively. It indicates that the weak arm-ECG in the proposed highly wearable SBP monitoring system can be a robust alternative of the strong chest-ECG. Besides, these three models own similar estimation performance, showing that the variables which highly correlates to the SBP, rather than the mechanisms used in the models, dominate the contribution. For the SBP estimated from chest-ECG and arm-PPG, a similar observation also holds.

### Further evaluation

As mentioned above, we also want to quantitatively evaluate the contribution of the introduction of HR to SBP model enhancement, therefore, we have implemented seven different PTT-SBP models for comparison purpose. The testing performance for models 1–7 is also given in Table 2. These seven models own similar estimation performance, indicating that their performance is mainly determined not by model assumptions but by the same variable, i.e., PTT which is correlated to the SBP. As shown in Table 2, the performance of these seven models is worse than the PTT&HR-SBP models. One thing worth noting is that the ME value usually cannot fully represent the model performance, since the positive and negative errors may cancel each other out and result in a relatively smaller ME. Therefore, we also introduce STD, MAE and RMSE to more effectively evaluate the model performance. Take the model 1 as an example, the ME ± STD, MAE and RMSE are 1.17 ± 6.00, 4.73 and 6.09 mmHg, respectively. Compared with model 10, the STD, MAE and RMSE for model 1 increase by 35.1, 28.5 and 29.3%, respectively. This quantitative comparative analysis shows that the PTT&HR-SBP models are much more robust than the PTT-SBP models, benefitting from the additionally introduced variable, i.e., HR.

**Fig. 7:**
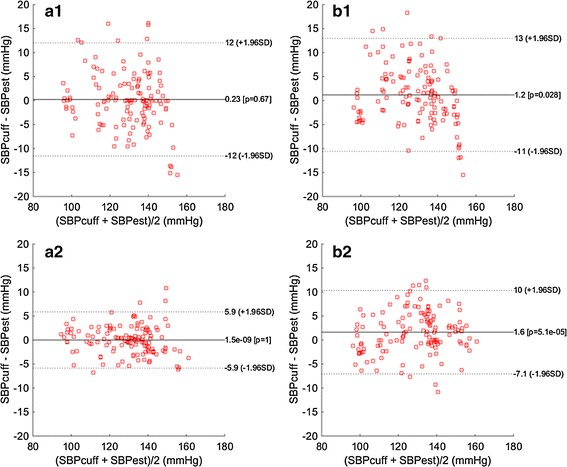
Training (**a1**, **a2**) and testing (**b1**, **b2**) performance of model 1 and 10 in terms of Bland–Altman plot, showing that (1) the testing performance on the unseen fresh testing data on the *right side* is worse than the training performance, and (2) PTT&HR-SBP model 10 is superior to PTT-SBP model 1 showing the necessity to include the HR information in SBP modeling

To further illustrate the different ability of PTT-SBP and PTT&HR-SBP models, a visualized performance comparison using Bland–Altman plots is given in Fig. 7. The top part (Fig. 7a1, b1) corresponds to the PTT-SBP model 1, and the bottom part (Fig. 7a2, b2) corresponds to the PTT&HR-SBP model 10. Moreover, not only testing results but also training results of model 1 and 10 are compared in Fig. 7, to fully illustrate their ability in model fitting and model generalization. In terms of the training performance, model 10 (Fig. 7a2) greatly outperforms model 1 (Fig. 7a), indicating that the introduction of the HR information helps fit the SBP model more effectively onto the training data. When it comes to the testing performance, the Bland–Altman plots for both model 10 (Fig. 7b2) and model 1 (Fig. 7b1) inevitably deteriorate compared with the Bland–Altman plots corresponding to the training performance, due to applying the fitted models onto the unseen fresh testing data. However, model 10 is stilly obviously more robust than model 1, based on the observation that the former one owns a better scatter of the estimation differences, i.e., its distribution is more concentrated in the low error area than the latter one. This visual illustration shows that the PTT&HR-SBP model 10 outperforms the PTT-SBP model 1 in terms of both model fitting and model generalization ability. It indicates that further introduction of appropriate variables in SBP model improvement is highly necessary to more effectively represent the underlying complex blood pressure generation and propagation mechanisms [2].

## Discussion

### Key considerations

As one of the key enabling factors in effective hypertension and heart health management, ubiquitous SBP and HR monitors have been attracting tremendous attentions [1], but at the same time, still face several major impediments, such as poor wearability, lack of widely accepted robust SBP models, insufficient proofing of the generalization ability of the fitted SBP models, etc. Focusing on these challenges, many efforts have been made in this study.

It is well known that wearability is directly related to the acceptance degree of the long-term SBP and HR monitors. The traditional approaches such as the invasive catheterization method and the noninvasive cuff-based oscillometry method [2], are not suitable for long-term applications. For ECG and PPG-based wearable solutions, although many investigations have already proposed some sensors placement methods, they still suffer from some limitations in terms of the comfortableness and convenience. For example, to collect the ECG signal, the chest placement has been used in many studies [3, 22], since the electrodes are so close to the heart that a strong ECG signal can be easily obtained, which simplifies the processing algorithms and brings a high motion-artifact tolerant ability. However, the chest electrodes placement may induce uncomfortableness, since a chest strap is usually needed to surround the body to fix the sensors and attach them to the skin. Another uncomfortableness may come from the sweat during long-term applications, especially in hot weather. Besides, some works apply a double-wrist-ECG plus finger-PPG setup [4], which prevents a chest strap, but usually needs an additional wire to collect the skin potential difference from two distant sites, which is highly inconvenient in daily applications. This limitation is also critical in overnight applications since the wire may impact the sleep quality. Of course, an extra wireless module may replace the wire, but inevitably introduces more hardware cost. An interesting alternative is using the non-contact ECG electrodes on the T-shirt which may be both comfortable and convenient, however, one critical challenge of this method is that it usually severely suffers from motion artifacts, meaning that the stretch of cloth or a vertical movement between the skin and the cloth may significantly impact or corrupt the ECG signal [23]. Another thing worth noting is that the PPG sensor may also introduce uncomfortableness with a chest placement method, or pose more challenges to the integration of PPG and ECG sensors with a double-wrist-ECG plus finger-PPG placement approach [4].

To achieve high comfortableness and convenience, we propose a single-arm placement approach, with both ECG and PPG sensors integrated into one arm band. This method avoids the chest strap and the additional wire or wireless module, providing a high level of integration by putting all sensors into one band. On the other hand, the single-arm placement for the ECG signal acquisition is highly challenging, since the reference/signal electrodes are too close to each other and in a similar direction referring to the heart, resulting in a very weak potential difference. By maximizing the distance between these two electrodes, i.e., placing one of them on the top side of the left arm and the other on the bottom side, and reducing the distance between the heart and the electrodes, i.e., putting the electrodes on the upper arm not the forearm, a weak but still distinguishable ECG signal is finally achieved, with an amplitude around only 10% of that of the chest-ECG signal. The acquired weak arm-ECG signal is successfully used in heartbeat locations identification, PTT measurement, and heart rate estimation which has a high correlation with the estimates based on the chest-ECG signal.

Another crucial consideration is the model applied to estimate the SBP. Although there are a large number of diverse SBP models, many efforts are still being made to further enhance the robustness and it is hard to select out one model which can well fit all application scenarios. In our study, we choose PTT&HR-SBP model to perform the SBP estimation task, meanwhile, we have also implemented PTT-SBP models to make a comparative analysis. In total, ten different SBP models [2, 3, 6, 7, 17–19] are taken into account in terms of the SBP estimation based on the arm-ECG/PPG signals. These models are built upon different mechanisms. For example, the SBP was assumed to be an inverse linear function of the PTT based on the fact that the propagation time of the mechanical pressure wave is reversely related to the SBP [2]. Another work assumed the SBP to be an inverse quadrative function of the PTT based the combined action of the pulse wave and the energy of wave (kinetic and the gravitational potential energy) [18]. Also, the SBP was regarded as a linear function of both PTT and HR since the observations showed that both PTT and HR correlate to the SBP [6].

To thoroughly compare these SBP models and illustrate the effectiveness of applying the PTT&HR-SBP model to the SBP estimation task, four major considerations are made. Firstly, seven PTT-SBP models without HR embedded are compared with three PTT&HR-SBP models with HR enhanced, to demonstrate that the HR information can significantly contribute to the robustness of the SBP model. A similar changing trend is observed on both HR and SBPcuff curves when perturbing the SBP with exercise stress, which confirms the previous observation that there is a high correlation between the HR and SBP [6]. Leveraging the HR information, the PTT&HR-SBP models greatly outperform the PTT-SBP models, as shown by the Bland–Altman plot in Fig. 7 and the performance summary in Table 2. Besides, the performance is similar among PTT-SBP models or PTT&HR-SBP models, which indicates that the complexity of the models has less contribution to the model improvement compared to HR, i.e., finding more SBP-related variables seems more significant than assuming diverse equations since the underlying accurate mechanism of SBP is highly complicated. Secondly, in model training, some models have already fixed some parameters in the equation based on empirical statistics, however, all the parameters are trainable in our study considering there is no empirical statistics for those parameters in single-arm scenarios, which is also to provide more general modeling of the SBP. Thirdly, the evaluation of the SBP models is mainly performed on the unseen fresh testing data, which is the key to show the generalization ability of the trained model. The testing performance in Table 2 indicates the fitted model can be well generalized to the data that has not been used to train the model. Finally, considering the single-arm-ECG signal is much weaker than the chest-ECG signal and thus sometimes sensitive to the motion artifacts, we also compare the single-arm-ECG signal with the chest-ECG signal in terms of the SBP estimation, to figure out whether the former one can robustly substitute for the latter one. The slightly different MAE and RMSE results between SBP estimation based on these two ECG placement methods shown in Table 2, indicate that the single-arm-ECG is both a highly convenient and robust alternative to the chest-ECG signal.

Based on the experimental results on the above key considerations on the SBP model, the PTT&HR-SBP models with HR enhanced can provide a more robust SBP estimation ability compared with the PTT-SBP models, and the trained models own a good generalization ability to the unseen testing data.

## Limitations and future work

Our current study mainly focuses on challenges discussed above in wearable SBP and HR monitoring, for subjects without heart diseases. Long-term wearable SBP and HR tracking for these users are also highly significant, because valuable immediate and statistical information can be provided for better health management and fitness monitoring such as evaluating stress, anxiety, sport performance and daily activities [24–26].

However, it is also highly necessary to further explore how to effectively estimate the SBP and HR with sing-arm signals for subjects with diverse health conditions, such as atrial fibrillation (AFib) which is a common arrhythmia and results in irregular heartbeat patterns [2]. When irregular heartbeats exist, the single-arm-based solution is more challenging than two-finger-based ones such as AliveCor which is for heart rate and early AFiB detection [27]. This is because the arm-ECG is much weaker due to a non-standard but highly wearable ECG lead configuration. In future, we will study how to robustly estimate SBP and HR for subjects with diverse health conditions to expand the applications scenarios of the single-arm-based easy-worn monitor.

Moreover, our current study is expected to illustrate the feasibility of the highly wearable single-arm blood pressure monitoring system. We have evaluated the proposed system on the data collected over a short duration and when subjects took a single sitting position, which is usually the strategy used in many wearable blood pressure monitoring studies [3]. Meanwhile, there are a few works focusing on the blood pressure estimation over a longer duration, such as days or months, and proposing some model recalibration techniques, or focusing on the influence of different body postures on the blood pressure, which show that it may be necessary to further include body posture information in the blood pressure modelling [3]. Our future research will also include recalibrating the blood pressure model over a longer duration and studying the influence of diverse body postures on the blood pressure modelling.

Besides, we will further evaluate the proposed system on more single-arm-ECG/PPG data acquired. Incorporating the signal quality information to dynamically refine the SBP estimates may also be helpful since the sensor-skin contact status is usually time-varying and has dynamic impacts on the signals acquired [28]. We are also interested in continuously improving the signal quality of the acquired weak single-arm-ECG signal such that it can provide a clearer morphology with well-formed P wave and T wave for medical interpretation purpose [29, 30].

## Conclusions

In this paper, we propose a wearable cuff-less system for long-term daily blood pressure and heart rate monitoring applications. To enhance the wearability, the ECG and PPG sensors are all integrated into a single-arm band. For arm-ECG acquisition, a non-standard single-lead configuration is provided, which is much more convenient and comfortable than normal wearable ECG placements, such as putting the electrodes on the chest (ECG and PPG), or two wrists (ECG) plus one finger (PPG). A weak arm-ECG signal with an amplitude only around 10% of the chest-ECG signal, is successfully obtained by our newly established hardware prototype. This weak arm-ECG signal is then used for heartbeat location identification and heart rate estimation with a machine learning-enabled framework. The HR estimation has a mean absolute error (MAE) and a root mean square error (RMSE) of 0.21 and 1.20 beats per min (BPM), respectively. Leveraging both arm-ECG and arm-PPG signals, the pulse transit time (PTT) information is then achieved and applied to systolic blood pressure (SBP) estimation.

The PTT&HR-SBP model is applied for SBP estimation considering both PTT and HR are correlated with SBP. Meanwhile, to thoroughly compare various SBP estimation approaches, ten SBP models based on diverse mechanisms are considered, such as applying a linear, quadratic or exponential equation, or leveraging the heart rate (HR) information to enhance the SBP model. Experimental results show that three PTT&HR-SBP models significantly outperform seven PTT-SBP models, indicating the necessary to introduce the HR information in robust SBP estimation. Moreover, the trained SPB models based on the arm-ECG/PPG signals are evaluated on the unseen data to show their high generalization ability. The estimated SBP is comparable to the estimates based on chest-ECG/arm-PPG signals, meaning that the arm-ECG signal can effectively replace the chest-ECG signals in terms of robust SBP estimation. Specially, for the PTT&HR-SBP models, the SBP estimation performance is 1.63 ± 4.44, 3.68, 4.71 mmHg in terms of ME ± STD, MAE and RMSE, respectively. In summary, the proposed wearable cuff-less SBP and HR monitoring system is expected to enable highly convenient SBP and HR monitoring applications and contribute to ubiquitous long-term hypertension, heart health and fitness management.

## Abbreviations

BP: blood pressure
SBP: systolic blood pressure
HR: heart rate
ECG: electrocardiography
PPG: photoplethysmogram
EMG: electromyography
ICG: cardiography
PCG: phonocardiogram
PTT: pulse transit time
PTT-SBP: PTT-based SBP model
PTT&HR-SBP: PTT and HR-based SBP model
PEP: pre-ejection period
ADC: analog-to-digital converter
ADC: microcontroller
MLEF: machine learning-enabled framework
IHR: instantaneous heart rate
MAE: mean absolute error
RMSE: root mean square error
SD: standard deviation
BPM: beats per minute
